# Tandem gene duplication and recombination at the *AT3* locus in the Solanaceae, a gene essential for capsaicinoid biosynthesis in *Capsicum*

**DOI:** 10.1371/journal.pone.0210510

**Published:** 2019-01-23

**Authors:** Ashley N. Egan, Shanna Moore, Giulia Marina Stellari, Byoung-Cheorl Kang, Molly M. Jahn

**Affiliations:** 1 Computational Biology Institute, George Washington University, Ashburn, Virginia, United States of America; 2 Department of Physics, Howard Hughes Medical Institute, Cornell University, Ithaca, New York, United States of America; 3 Department of Plant Biology, Cornell University, Ithaca, New York, United States of America; 4 Department of Horticulture, Seoul National University, Seoul, Republic of Korea; 5 Department of Agronomy, University of Wisconsin-Madison, USDA FPL, Madison, Wisconsin, United States of America; University of Tsukuba, JAPAN

## Abstract

Capsaicinoids are compounds synthesized exclusively in the genus *Capsicum* and are responsible for the burning sensation experienced when consuming hot pepper fruits. To date, only one gene, *AT3*, a member of the BAHD family of acyltransferases, is currently known to have a measurable quantitative effect on capsaicinoid biosynthesis. Multiple *AT3* paralogs exist in the *Capsicum* genome, but their evolutionary relationships have not been characterized well. Recessive alleles at this locus result in absence of capsaicinoids in pepper fruit. To explore the evolution of *AT3* in *Capsicum* and the Solanaceae, we sequenced this gene from diverse *Capsicum* genotypes and species, along with a number of representative solanaceous taxa. Our results revealed that the coding region of *AT3* is highly conserved throughout the family. Further, we uncovered a tandem duplication that predates the diversification of the Solanaceae taxa sampled in this study. This pair of tandem duplications were designated *AT3-1* and *AT3-*2. Sequence alignments showed that the *AT3-2* locus, a pseudogene, retains regions of amino acid conservation relative to *AT3-1*. Gene tree estimation demonstrated that *AT3-1* and *AT3-2* form well supported, distinct clades. In *C*. *rhomboideum*, a non-pungent basal *Capsicum* species, we describe a recombination event between *AT3-1* and *AT3-2* that modified the putative active site of *AT3-1*, also resulting in a frame-shift mutation in the second exon. Our data suggest that duplication of the original *AT3* representative, in combination with divergence and pseudogene degeneration, may account for the patterns of sequence divergence and punctuated amino acid conservation observed in this study. Further, an early rearrangement in *C*. *rhomboidium* could account for the absence of pungency in this *Capsicum* species.

## Introduction

Capsaicinoids, the alkaloids that confer pungency, or ‘heat’, to pepper fruits are produced exclusively by species of the genus *Capsicum* L. (Solanaceae), presumably evolving to deter mammalian herbivory [1, 2]. Pungency is one of the most important characteristics of *Capsicum* fruit, and a reason the fruits are prized throughout the world. The best-known cultivated varieties are *C*. *annuum* L. (e.g. bell types and hot peppers such as jalapeno and cayenne), *C*. *frutescens* L. (Tabasco) and *C*. *chinense* Jacq. (Habanero). Capsaicinoids have also been utilized historically as an analgesic [3], and more recently to treat neurological disorders, bladder and digestive syndromes, and cancer [4]. The numerous uses and applications of capsaicinoids have inspired scientific and commercial research efforts to improve our understanding of capsaicinoid biosynthesis.

Efforts to clarify the biosynthesis of capsaicinoids have resulted in the cloning and characterization of the *Pun1* locus. *Pun1* (formerly known as *C*) was first documented in the early 1900s and, in its recessive form, is epistatic to all other pungency related loci [5, 6]. *Pun1* has significant homology to the BAHD family of acyltransferases (named for the first four characterized members of the family BEAT, AHCT, HCBT, DAT) and thus was designated *AT3* [6, 7]. Members of the BAHD family catalyze the transfer of the acyl moiety to a wide range of acceptor molecules and are essential in the biosynthesis of a large array of natural plant compounds such as lignin, phenolics, alkaloids, anthocyanins and volatile esters [8]. Specifically, *AT3-1* lies within clade III of the BAHD family. Many of the representative genes in this clade can accept a diverse range of alcohol substrates, however most utilize acetyl-CoA as the major acyl donor. Given the importance of this pathway and the lengthy history of investigation, it is striking that the substrate, specificity, and enzymatic function of *AT3* in the capsaicinoid biosynthetic pathway remains unknown, although all evidence to date indicates that *AT3* is essential for capsaicinoid biosynthesis [9–11].

Our understanding of the essential nature of *AT3* to capsaicinoid biosynthesis has largely been improved through studies of non-pungent peppers. Stewart et al. [6] demonstrated that in non-pungent variants of *C*. *annuum*, *AT3* is present but includes a large 2.5 kb deletion in the 5’ end of the gene that resulted in the absence of any detectable transcript or protein. This state precisely correlated with absence of capsaicinoids and, ultimately, the loss of pungency in fruit. In a subsequent study, an additional and distinct truncated *AT3* allele was reported in a cultivar of *C*. *chinense*, that also resulted in the loss of pungency [12]. While the evidence that *AT3* is essential for capsaicinoid biosynthesis is compelling, and it is clear that major truncations in paralogues of this gene have been correlated with loss of pungency in at least two *Capsicum* species, many questions remain about the precise structure and function of this gene in the Solanaceae.

The pivotal, but still ambiguous, role of *AT3* in capsaicinoid biosynthesis prompted the current study of the evolution of this gene family within the genus *Capsicum* in the context of a sampling of this gene and related gene family members across other genera in the Solanaceae. We sequenced *AT3* from multiple pepper species and cultivars, with additional representative species from a diverse panel of solanaceous genera. Surprisingly, in this study, we found that *AT3* shows a significant level of conservation throughout the Solanaceae, while also showing evidence of expansion via tandem gene duplication, a class of rearrangement well known in eukaryotes [13]. Ohno [14] posited that duplicated genes constituted the raw material for evolution of novel traits because relaxed selection on one of the copies could lead to the evolution of novel functions, or neo-functionalization. In the ensuing nearly fifty years, many different types of evolutionarily significant outcomes from gene duplication events have been described, including neofunctionalization, subfunctionalization, and sub-neofunctionalization, where initial preservation of gene function by partitioning of expression leads to subsequent acquisition of novel function [15–17]. In addition, the proliferation of homologous gene copies via duplication provides increasing possibilities for recombination or gene conversion events, thus enabling diversification of homologs at the molecular level. The genus *Capsicum* presents a unique study system in the context of other solanaceous genera to investigate the evolutionary ramifications and impacts of gene duplication on the role of *AT3* in capsaicinoid biosynthesis.

We present here the cloning and characterization of a gene duplicate of *Pun1/AT3*, and a paralogue that we have designated *AT3-2*. We demonstrate that these duplicated genes which we have designated *AT3-1* and *AT3-2* represent ancient paralogous gene lineages whose duplication predates the diversification of solanaceous taxa we sampled. Further, this paper reports for the first time a recombination event in *C*. *rhomboideum* (Dunal) Kuntze revealing a putative basis for non-pungency in what is often seen as a basal and non-pungent species of the *Capsicum* genus. This new information contributes to our understanding of the evolution of the characteristic of pungent fruits in the genus *Capsicum*, and provides an explanation for the uniform absence of pungency in a basal lineage, *C*. *rhomboideum*. Finally, the complexity of the structural rearrangements between *AT3-1* and *AT3-2* provide insight regarding the origin(s) of pungency in *Capsicum* and the nature of variants in generally pungent species that may lack pungency. More precise knowledge of the structure and function of *AT3*, its effects on phenotype, and variant alleles found in this globally treasured genus will further illuminate evolution of this famous plant trait and facilitate efforts to manipulate the capsaicinoid pathway for specific, and perhaps novel, capsaicinoids.

## Materials and methods

### Plant growth and tissue collection

*Capsicum* germplasm, consisting of a set of stable lines that have been previously characterized and reported, was selected from a broad collection to represent varieties/species with commercial and/or research importance [6, 7, 18, 19]. Remaining Solanaceae germplasm was previously reported [18, 20]. All accessions from the species *Capsicum annuum*, *C*. *frutescens*, *C*. *chinense*, *C*. *chacoense* Hunz., *C*. *rhomboideum*, *Lycianthes dejecta* (Fernald) Bitter, *Solanum lycopersicum* L., *Solanum melongena* L., *Solanum pennellii* Correll, *Nicotiana benthamiana* Domin, *Datura stramonium* L., and *Petunia axillaris* subsp. *parodii* (Steere) Cabrera were grown in a greenhouse in Ithaca, NY from single seed selections or obtained from collaborators with well established, uniform and stable species as described in the references above. Growing conditions were approximately 27°/18°C (day/night) with a daily fertilization with Excel solution (200 ppm; The Scotts Company, Marysville, Ohio). Green (21 days post-anthesis, dpa) and ripe fruit (50 dpa) were collected for all pepper varieties. Mature green (35 dpa) and ripe fruit (breaker plus 10 days) were collected for *S*. *lycopersicum*. Age-matched fruit were collected for *Solanum pennellii* as well as immature and mature fruit from *S*. *melongena*. Upon harvest, all fruits were acclimated for 4–8 h in the laboratory. Seeds were harvested and pericarp tissue was immediately frozen in liquid nitrogen, ground to a powder, and stored at –80°C.

### RNA extraction & DNA isolation

RNA was extracted from all tissue types using Qiagen RNeasy (Qiagen, Valencia CA) per manufacturer’s instructions for isolation of plant and fungal RNA. For each extraction, approximately 50 ng of frozen, ground tissue was utilized and the final elution volume was 35 μl of RNase free water. RNA was denatured and visualized on an agarose gel. Quantity was assessed via spectrophotometer reading. Expanding leaf tissue was either used immediately for DNA extraction or frozen in liquid nitrogen and stored at -80°C for later extraction. Genomic DNA was extracted as described previously [21].

### Isolation and sequence analysis of *AT3*

Ten Bacterial Artificial Chromosomes (BACs) from a *C*. *frutescens* 2814 pBeloBACII DH10B library were identified as part of a previous study as positive for *AT3-1* (K. Liu and M Jahn, unpublished results). These BACs were further screened for the presence of the additional *AT3-2* intron fragment. Of those containing the additional *AT3-2* intron fragment, one (159M5) was selected at random and a large-scale DNA prep was done using Qiagen Midi Prep Kit (Qiagen, Valencia, CA) following the manufacturer’s instructions for very low copy plasmids.

*AT3-2*-unique primers were designed from the intron sequence (*AT3-2* INTRON F 5’–AAGTAAACTGAATTTGTTTCAAAA-3’; *AT3-2* INTRON R 5’ ATTTACCCTACATTATTATCGGTC– 3’, 364 bp product). These primers were used in conjunction with Bio S&T APAgene Gold Genome Walking Kit (BioS&T, Montreal, Canada), per manufacturer’s instructions, to isolate a full-length genomic sequence. Two separate sets of reactions were undertaken using either *AT3-2* INTRON F or *AT3-2* INTRON R as the GSPa primer.

Products were excised and gel purified using the Qiagen QIAquick Kit (Qiagen, Valencia, CA) according to the manufacturer’s instructions. The blunt end products were prepared for TA cloning as follows: 19.55 μl of gel purified PCR product, 1.5 μl 25 mM MgCl_2_, 1.25 μl 10mM dNTPs, 2.5 μl 10x Buffer, 0.2 μl Roche Taq (Roche, Indianapolis, IN). This was incubated at 72°C for 20 minutes and then ligated using the Invitrogen TOPO TA Cloning Kit (Invitrogen Corporation, Carlsbad, CA) for sequencing with the PCR4 vector and TOPO10 electro-competent cells. Ligations were performed as follows: 2 μl of gel purified PCR product, 3 μl water, 0.5 μl dilute salt (1:4 dilution of Salt Solution provided with kit), 0.5 μl TOPO vector; incubated at RT for 15 minutes and stored at -20°C. Two μl of ligation product was combined with TOPO10 cells, electroporated, combined with 500 μl 2xLB, incubated at 37°C for 30 minutes, then 300μl was plated on LB/agar/kanamyacin plates and incubated at 37°C overnight. Resulting colonies/plates were stored at 4°C.

Multiple clones from each ligation were selected into 3 mls of LB/kanamyacin and incubated with 200 rpm shaking at 37°C overnight. Plasmids were purified for sequencing using the QIAGEN QIAprep Kit (Qiagen, Valencia, CA) according to the manufacturer’s instructions. Sequencing was conducted by the Bioresources Center, Cornell University (www.brc.cornell.edu). Reactions were submitted to the sequencing facility as follows: 3 μl of resulting miniprep DNA, 1 μl 10mM M13F or R, 14 μl of water.

Cloned sequences were trimmed of vector and aligned with other sequences from the same ligation reaction using Seqman II 6.1 of DNASTAR suite of analysis software to generate consensus sequences. Overlapping sequences from the 5’ and 3’ end of *AT3-2* were assembled and aligned with existing *AT3-1* sequence for comparison. Subsequent *AT3-2* sequence was amplified utilizing two separate primer combinations to ensure amplification of *AT3-2* and not *AT3-1*: *AT3-2* F (5’–ATGGCTTTTGCATTGGTATCATCACCAT -3’) / AT3-2 INTRON F resulting in a 1.1 kb product and AT3-2 INTRON R/ AT3-2 R (5’- CGGTATACTCATTCTTACAGGTTT-3’) resulting in a 860 bp product. All amplifications were done in a 15 μl total volume (8.65 μl water, 1.5 μl 10x buffer, 1.0 μl 10mM dNTPs, 0.3 μl 10mM Primer F, 0.3 μl 10mM Primer R, 0.5 μl Stratagene Easy A Taq) and under the following conditions: 95°C for 5 minutes, 35 cycles of 95°C for 30 seconds, 65°C for 30 seconds 72°C for 1 minute, 72°C for 10 minutes, 4°C. Reactions were conducted using a PTC 225 Peltier Thermal Cycler (MJ Research, Watertown, MA).

### *AT3* expression analysis by northern blot and RT-PCR

To explore potential expression of *AT3* within our plant accessions, 10 micrograms of total RNA for each of the following tissues were prepared, blotted and hybridized as described previously [20]: *S*. *lycopersicum* cv Ailsa Craig leaf, flower, 35 dpa fruit, 50 dpa fruit; *C*. *chinense* cv Habanero 21 dpa fruit; *S*. *pennellii* Leaf, flower, 35 dpa fruit, and 50 dpa fruit. Filters were hybridized to a 32-P labeled full length *AT3-1* PCR product. We also used RT-PCR to examine whether a functional assay could differentiate between *AT3-1* and *AT3-2* expression. RT- PCR was done using primers that spanned the *AT3-1/AT3-2* intron and the Superscript II one step RT-PCR system according to the manufacturers protocols (Thermo Fisher Scientific, Massachusetts, US). Resulting products were visualized on agarose gels and then, due to significant sequence homology, resulting products were digested using six different restriction enzymes (EcoRV, BfaI, Tsp5091, Fnu4HI, AccI, and MnII) according to enzyme manufacturer protocols (New England Biolabs, Massachusetts, US) and visualized on agarose gels in an attempt to discern between *AT3-1* and *AT3-2*.

### DNA sequence alignment for downstream analyses

The *AT3* sequences produced as part of this work (GenBank accession numbers FJ687524-FJ687531, FJ755160-FJ755164, FJ755166-FJ755176, MF142764) were augmented with a number of other *AT3* gene family members across the Solanaceae gathered via BLAST searches against NCBI carried out in April 2017. *Capsicum annuum* genes annotated as *Pun1*, *BAHD*, *Catf1*, and *Catf2* were also included. A number of homologous genes from *Nicotiana* and *Solanum* and other genera that represent the broader AT3 gene family were included as outgroups, mostly designated as acylsugar acyltransferase and acylsugar acyltransferase-3like, assembled into a dataset incorporating 64 genes (Table 1). This coding dataset was aligned using MUSCLE [22] via the EMBL-EBI webserver [23] and honed to correct reading frame by eye using nucleotide and translated view within Ali-View [24]. Intronic regions within *AT3-1* and *AT3-2* were aligned separately for each paralog within Aliview using MUSCLE. The introns of *AT3-1* and *AT3-2* were initially excluded from the alignment, as they are non-homologous and caused a breakdown of the alignment of the flanking sequence when included. The introns of *AT3-1* and *AT3-2* were defined using the intron-exon boundary known from cloning the mRNA of *AT3-1* from *C*. *chinense* ‘Habanero’ [6]. Intronic regions were analyzed for gene tree estimation separately due to non-homology.

**Table 1:** List of NCBI accessions for sequences involved in this work.

**Sequences generated during this study**			
**Species**	**Cultivar/ecotype/ other designation**	**Gene notation**	**NCBI #**
*Capsicum annuum*	RNaky	*AT3-1*	FJ755173*
*Capsicum annuum*	Thai Hot	*AT3-1*	AY819029*
*Capsicum frutescens*	Tabasco	*AT3-1*	FJ755174*
*Capsicum frutescens*	PI594141 Pungent	*AT3-1*	FJ755175*
*Capsicum frutescens*	BG2814-6	*AT3-1*	AY819026
*Capsicum chinense*	Habanero	*AT3-1*	AY819027*
*Capsicum chacoense*	Non-pungent	*AT3-1*	FJ755176*
*Capsicum rhomboideum*	T70	*AT3-1* recombinant	MF142764**
*Lycianthes dejecta*		*AT3-1*	FJ755172*
*Solanum pennellii*		*AT3-1*	FJ755168*
*Nicotiana benthamiana*		*AT3-1*	FJ755171*
*Solanum lycopersicum*	Ailsa Craig	*AT3-1*	FJ755166*
*Solanum melongena*	Ichiban	*AT3-1*	FJ755170*
*Datura stramonium*		*AT3-1*	FJ755167*
*Petunia axillaris*		*AT3-1*	FJ755169*
*Capsicum annuum*	RNaky	*AT3-2*	FJ687524*
*Capsicum annuum*	Maor	*AT3-2*	FJ755161*
*Capsicum annuum*	Thai Hot	*AT3-2*	FJ687530*
*Capsicum frutescens*	BG2814-6	*AT3-2*	FJ687526*
*Capsicum frutescens*	Tabasco	*AT3-2*	FJ755160*
*Capsicum frutescens*	PI594141 Pungent	*AT3-2*	FJ687527*
*Capsicum frutescens*	BG2814-6	*AT3-2*	FJ755162*
*Capsicum chinense*	Habanero	*AT3-2*	FJ755163*
*Capsicum chacoense*		*AT3-2*	FJ755164*
*Capsicum rhomboideum*	T70	*AT3-2*	FJ687529*
*Lycianthes dejecta*		*AT3-2*	FJ687528*
*Solanum pennellii*		*AT3-2*	FJ687531*
*Nicotiana benthamiana*		*AT3-2*	FJ687525*
**Sequences from NCBI based on *AT3-1* or *AT3-2* homology**			
**Species**	**Cultivar/ecotype/ other designation**	**Gene notation**	NCBI #
*Capsicum annuum*	Yidu-Red	Pun1	GU300812
*Capsicum annuum*		acyl sugar acyltransferase 3 like	NM_001324769
*Capsicum annuum*		catf1	AB206919
*Capsicum annuum*		catf2	AB206920
*Capsicum annuum*	Zunla-1	acylsugar	XM_016704776
*Capsicum annuum*	Sweet 3575	Pun1	AY819032
*Capsicum annuum*	Hot 1493	Pun1	AY819028
*Capsicum annuum*			GD123213
*Capsicum annuum*			GD120313
*Capsicum annuum*		acyl sugar acyltransferase 3 like	NM001324769
*Capsicum annuum*		acyl sugar acyltransferase 3 like	XM016704776
*Capsicum chacoense*	PI260433-p	BAHD	FJ871984
*Capsicum chinense*	NMCA30036	Pun 1–2	EF104910
*Capsicum frutescens*	Shuanla	Pun1	HM854860
*Capsicum frutescens*	Cakra Hijau	AT3	GD123213
*Capsicum frutescens*	Non-pungent	BAHD	**FJ871985**
*Capsicum frutescens*	BG2814-6	Pun1–1	AY819026
*Capsicum frutescens*	Shuanla	Pun1-1	HM854860
*Nicotiana benthamiana*			CK283402
*Nicotiana sylvestris*		deacetylvindoline O-acetyltransferase-like	XM009759601
*Nicotiana tabacum*		acyl sugar acyltransferase 3 like	XM016624127
*Nicotiana tabacum*		acyl sugar acyltransferase 3 like	XM016609395
*Nicotiana tabacum*		acyl sugar acyltransferase 3 like	XM016584740
*Nicotiana tabacum*		acyl sugar acyltransferase 3 like	XM016633955
*Nicotiana tabacum*		acyl sugar acyltransferase 3 like	XM009606501
*Nicotiana tabacum*		acyl sugar acyltransferase 3 like	XM009606502
*Nicotiana tabacum*		acyl sugar acyltransferase 3 like	XM016618628
*Nicotiana tabacum*		acyl sugar acyltransferase 3 like	XM016598426
*Nicotiana tabacum*		acyl sugar acyltransferase 3 like	XM016648743
*Nicotiana tabacum*		acyl sugar acyltransferase 3 like	XM016612153
*Nicotiana tabacum*			FS433866
*Nicotiana tomentosiformis*		acyl sugar acyltransferase 3 like	XM009590484
*Petunia x hybrida*			FN004147
*Phyllostachys edulis*			FG395562
*Solanum habrochaites*		trichome EST	AW617268
*Solanum lycopersicum*	Heinz		AC215475
*Solanum lycopersicum*		acyl sugar acyltransferase 3 like	XM004232585
*Solanum lycopersicum*		acyl sugar acyltransferase 3 like	XM004232587
*Solanum lycopersicum*		acyl sugar acyltransferase 3 like	XM004232586
*Solanum pennellii*		acyl sugar acyltransferase 3 like	XM015210076
*Solanum pennellii*		acyl sugar acyltransferase 3 like	XM015209963
*Solanum pennellii*		acyl sugar acyltransferase 3 like	XM015209888
*Solanum pennellii*		acyl sugar acyltransferase 3 like	XM015209896
*Solanum tuberosum*		acyl sugar acyltransferase 3 like	XM006363120
*Solanum tuberosum*		acyl sugar acyltransferase 3 like	XM006363146
*Solanum tuberosum*		acyl sugar acyltransferase 3 like	XM006363147
*Solanum tuberosum*		acyl sugar acyltransferase 3 like	XM015303315
*Solanum tuberosum*		acyl sugar acyltransferase 3 like	XM006363121

**Bold** designates those sequences produced for this work but published previously.

* used in initial alignment;

** new sequences published herein.

### Analysis of recombination

Given the unexpected association between *C*. *rhomboideum AT3-1* with the *AT3-2* clade during preliminary phylogenetic analyses, the alignment was tested for the presence of recombination events using the program RDP4 [25] with seven different methods under default settings: RDP [26], GENECONV [27], BootScan [28], MaxChi [29], Chimaera [30], SiScan [31], and 3Seq [32]. Only recombination signals detected by five or more methods were considered plausible. Statistical significance was assessed using a Bonferroni-corrected p-value threshold of P ≤ 0.05. The full-length alignment, containing the coding regions of AT3-1 and AT3-2 with exons 1 and 2 spliced together (designated ex1ex2) was scanned for recombinant sequences. Preliminary recombination analyses suggested that two sequences were acting as ‘wild card’ sequences: N_tomentosiformis_LOC104098383_XM_009605101 and Capsicum_frutescens_BAHD_FJ871985, which were removed from final recombination analyses and subsequent phylogenetic analyses. These sequences were disproportionately impacting recombination results and suggested by RDP4 to be products of “processes other than recombination.”

### Phylogeny estimation

Gene trees were estimated using Randomized Axelerated Maximum Likelihood (RAxML v8) [33] either within the RDP4 program or on the RAxML Blackbox webserver [34] using GTRCAT model of evolution. Nodal support was estimated through 100 bootstrap replicates. Gene trees were estimated on the full coding dataset comprising 71 sequences with an aligned length of 1299 bp (designated ex1ex2), on a dataset with recombinant sequences segregated into recombinant portions (and thus the T70 recombinant sequence represented twice in the dataset), on a reduced dataset optimized for use synonymous substitution analyses wherein gaps or missing data were minimized, comprising 44 sequences and an aligned length of 1287 bp (see below for further details), and on intronic regions for *AT3-1* and *AT3-2* and one each of *catf1* and *catf2*, comprising 35 sequences and an aligned length of 756 bp. Trees for the expanded dataset were rooted to the clade containing either the AT3 homolog in *Phyllostachys edulis* (Carriere) J.Houz. (FG395562) or to a distant AT3 homolog from *Solanum lycopersicum* L. (AC215475 gene start at position 46024) for the Ks dataset. Alternative phylogenetic trees were tested for statistical differences in topology using the Shimodaira-Hasegawa test (SH) [35] and the approximately unbiased test (AU) [36].

### *Ks* estimation

Synonymous (*Ks*) and non-synonymous substitution rates calculated across protein-coding genes can provide insights into relative rates of evolution, sequence conservation, signatures of positive selection, and dates of divergence via assumption of a molecular clock. By estimating and comparing *Ks* between *AT3-1* and *AT3-2* and the regions affected by recombination, we may glean information about the relative selection pressures and timing of events across this pair of gene duplicates. Synonymous and non-synonymous substitution rates were estimated for all pairwise comparisons of sequences within a reduced sample set in PAML v4 [37] using the YN00 method [38]. During *Ks* estimation, PAML removes columns of aligned data with any missing (gapped) characters. To maximize *Ks* output, all sequences that had long ‘indels’ and several outgroup sequences with significant missing data were removed, resulting in datasets with 44 sequences (S1 File). However, each major lineage or clade recovered in the full dataset phylogenetic analyses was represented by at least one sequence in the alignment used for *Ks* estimation, thus enabling estimates across all major internal nodes. *Ks* estimates were calculated for the full coding alignment (ex1ex2br) and alignments broken at the recombination breakpoints (ex1br and ex2br).

## Results

### Sequencing of *AT3-1* and *AT3-2*

**Fig 1:**
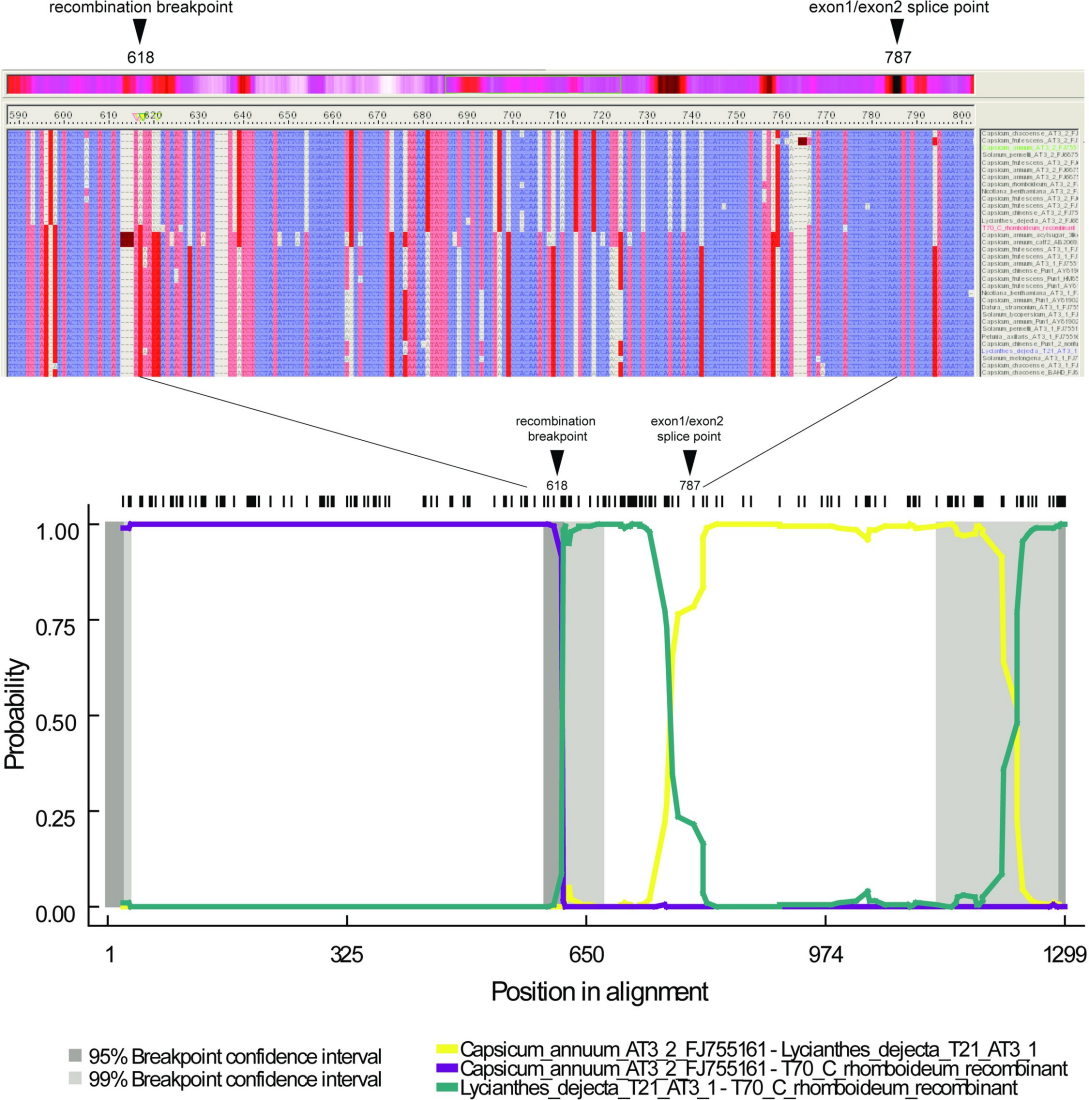
Diagram illustrating the recombination event between *AT3-1* and *AT3-2*. Upper plot shows a snapshot of a portion of aligned *AT3-1* and *AT3-2* showing the recombination breakpoint and exon1/exon2 splice boundary within the coding sequence alignment. The recombinant *C*. *rhomboideum* T70 sequence is in red lettering. The lower plot is a screen shot of the RDP4 pairwise similarity plot that depicts the pairwise identities among the recombinant and its putative parents. The black hash marks at the top of the plot indicate the positions of informative sites along the length of the alignment.

This work produced twelve *AT3-1* sequences of approximately 1630 base pairs comprising the 5’ UTR, the single intron, and the near full-length coding region (Table 1). All sequences showed a high degree of conservation at both the nucleotide and amino acid levels, as well as conservation of the intron/exon boundary (Fig 1). *Nicotiana benthamiana AT3-1* revealed a two-base pair insertion/deletion (indel) resulting in a frame-shift mutation that we predict would produce a truncated *AT3-1* protein.

In the course of cloning and characterizing *AT3-1* from *Capsicum* and other Solanaceae representatives, a novel intronic sequence was discovered that had no recognizable homology to *AT3-1*, yet shared the same intron-exon boundaries (Fig 1). This novel sequence was identified, together with *AT3-1*, in multiple clones of a Bacterial Artificial Chromosome (BAC) library made from *C*. *frutescens* BG2814-6. Using a single BAC, a genome walk across the intron out to the 5’ and 3’ ends of the gene revealed DNA sequence showing strong similarity to *AT3-1*. This tandem duplicate, which we have named *AT3-2*, was approximately 1600 base pairs in length and was subsequently cloned and sequenced from *Capsicum*, *Nicotiana*, *Datura*, *Solanum* and *Lycianthes* (Table 1).

Our study further revealed that this duplicated sequence, *AT3-2*, is also highly conserved throughout the Solanaceae. Numerous non-synonymous mutations relative to *AT3-1* are shared among all *AT3-2* sequences, consistent with the possibility that the duplication event that included the coding region of *AT3-2* occurred before subsequent speciation of the taxa represented in our study. The alignment of the first exon between *AT3-1* and *AT3-2* also includes indels not in multiples of three that disturb the open reading frame of *AT3-2* while maintaining punctuated regions of amino acid conservation relative to *AT3-1*. This observation is further consistent with the possibility that *AT3-2* is a pseudogene of *AT3-1*.

### *AT3* expression assessed via northern blots and RT-PCR

**Fig 2:**
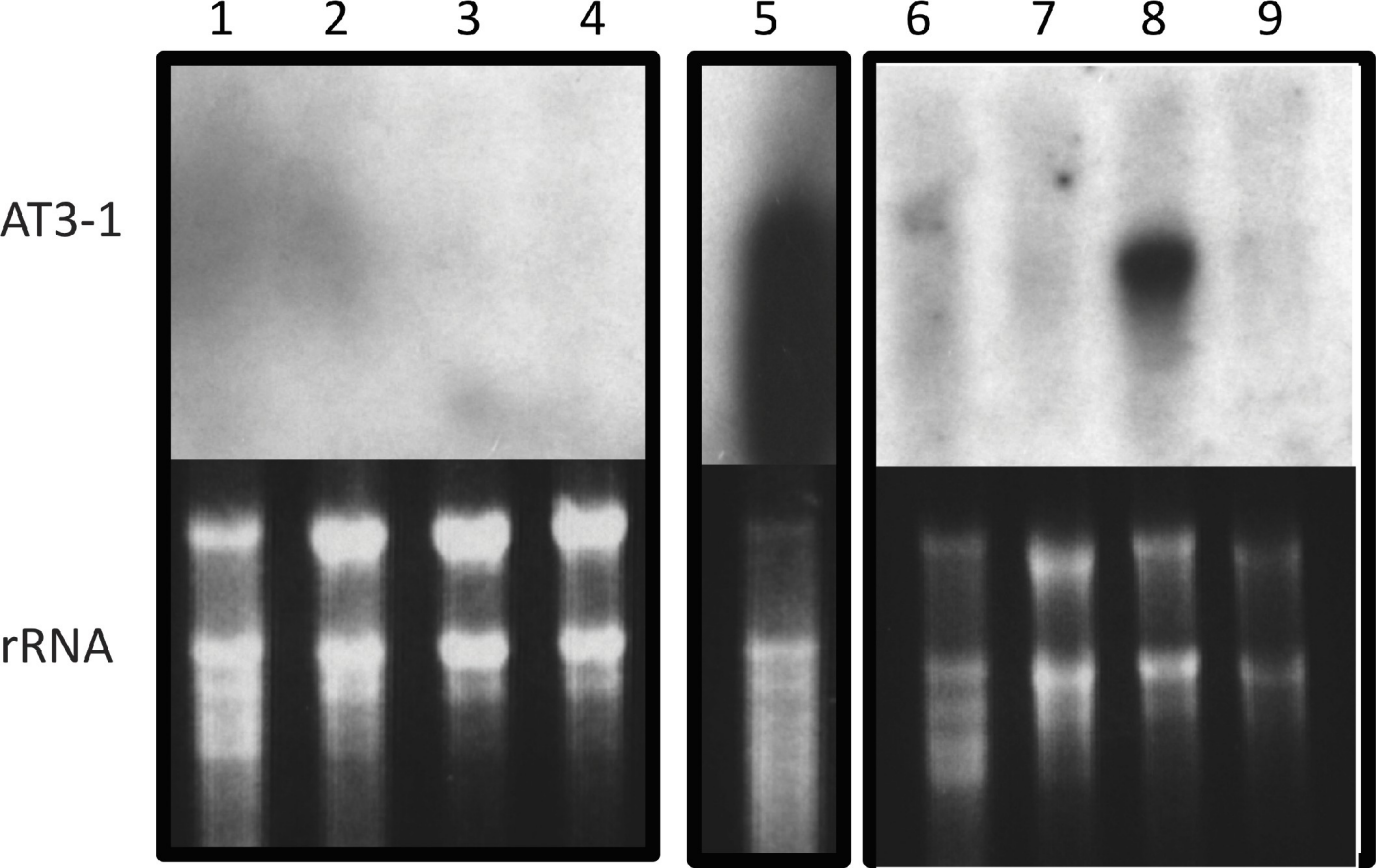
Differential expression of *AT3-1* in solanaceous species. A full length *AT3-1* probe was hybridized to RNA gel blots containing the following tissues: Lanes 1–4 *Solanum lycopersicum* cv Ailsa Craig 1. Leaf, 2. Flower, 3. Mature green fruit (21 days post anthesis), 4. Red ripe fruit (50 days post anthesis); 5 –*Capsicum chinense* cv Habanero fruit 21 days post anthesis; 6–9 *Solanum pennellii* 6. Leaf, 7. Flower, 8. Fruit 21 days post anthesis, 9. Fruit 50 days post anthesis.

In *Capsicum*, *AT3* expression is confined to the developing pepper placenta, shown previously by RNA gel-blot [6] and through RNA-seq analysis [9]. Based on the extensive sequence homology we observed throughout the Solanaceae, we further examined expression in a sampling of tomato species, as they experience similar fleshy fruit production. Utilizing a full-length cDNA as a probe, *AT3* expression was detected in 35 dpa fruit in *S*. *pennellii* (a wild tomato relative that does not ripen to red), but was not observed in age-matched *S*. *lycopersicum* (Fig 2). Habanero 21 dpa fruit was used as a positive control, as 21 dpa is when *AT3-1* expression becomes visible. The *S*. *pennellii AT3-1* expression was less than that seen in Habanero, as noted by the over-exposure of Habanero *AT3-1*. We then utilized RT-PCR to determine if a functional assay to differentiate between *AT3-1* and *AT3-2* was feasible. Unfortunately, despite using six different restriction enzymes, we were unable to conclusively determine, in any tissue examined, if *AT3-2* was expressed—owing either to the lack of expression of *AT3-2* or to significant sequence conservation that resulted in a very small potential size difference in any resulting fragments and the limited enzymes that might discern this potential difference. Given the sequence evidence for pseudogenization of *AT3-2*, a process that may preclude any expression, we ultimately concluded that any expression seen was very likely *AT3-1*.

### Recombination analysis

Six recombination detection methods implemented in RDP4 detected a strongly supported (corrected binomial probability p-value = 1.395 E -18: RDP 5.365 X 10^−18^; GENECONV 2.857 x 10^−13^; BootScan 1.395 x 10^−18^; MaxChi 6.557 x 10^−14^; Chimaera 5.524 x 10^−07^; 3Seq 2.870 x 10^−17^) recombination event involving *C*. *rhomboideum* as the recombinant (designated here as T70), with *Capsicum annuum AT3-2* FJ755161 as the major parent (sharing 92.6% similarity) and *Lycianthes dejecta AT3-1* FJ755172 as the minor parent (sharing 99.5% similarity). The higher sequence similarity of the minor parent, an *AT3-1* sequence, reflects the higher conservation of *AT3-1* relative to the pseudogenized *AT3-2* parent. The major and minor parents were localized to the *AT3-2* and *AT3-1* clades respectively. The above-designated parents showed the highest probability within the respective clades; however, other members of the clade may not show statistically significant differences in probability of parentage. The breakpoint regions are effectively at positions 1–618 and 619–1299 in the aligned coding ex1ex2 dataset. The relative association of *C*. *rhomboideum AT3-1* with *AT3-1* prior to the breakpoint, and with *AT3-2* after the breakpoint can roughly be identified by eye, as is evident in Fig 1.

### Phylogenetic analysis

**Fig 3:**
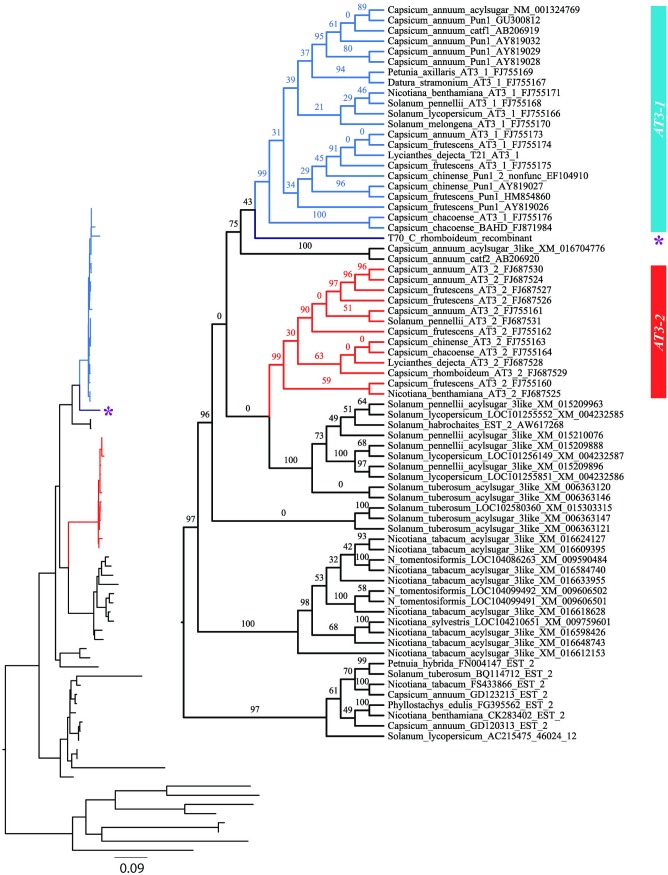
Maximum likelihood gene tree estimated from complete coding sequence alignment (ex1ex2) ignoring recombination. *AT3-1* branches are colored blue, *AT3-2* are red, the T70 *C*. *rhomboideum* recombinant is purple and designated with a star. Numbers above branches are bootstrap support values for corresponding nodes. Inset shows branch lengths.

Phylogenetic analysis of the entire coding sequence alignment (ex1ex2) without intronic sequences, also ignoring recombination, resolved two major clades representing *AT3-1* and *AT3-2* with strong support (BP = 99 for each; Fig 3). The *C*. *rhomboideum* recombinant sequence, T70, was resolved as sister to the *AT3-1* clade, but with low support (BP = 43), a result not unexpected given the disparate and conflicting phylogenetic signals within its length. Both *AT3-1* and *AT3-2* showed high levels of conservation as evidenced by the short branch lengths therein (Fig 3). Each clade included *Capsicum* and other Solanaceae genera, suggesting that the paralogs represent an ancient duplication that predated divergence of the family.

**Fig 4:**
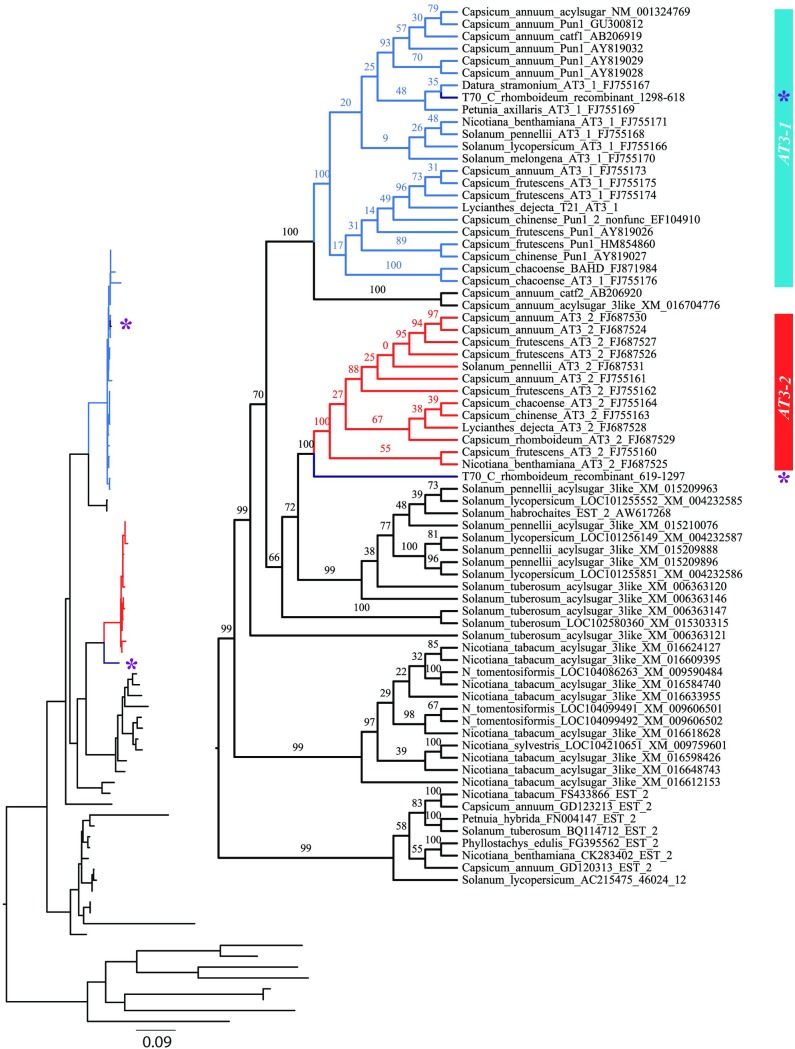
Maximum likelihood gene tree estimated from the complete coding sequence alignment (ex1ex2) with recombinant regions of *C*. *rhomboideum* separated. *AT3-1* branches are colored blue, *AT3-2* are red, the T70 *C*. *rhomboideum* recombinant portions are purple and designated with a star. Numbers above branches are bootstrap support values for corresponding nodes. Inset shows branch lengths.

Phylogenies constructed separately on each of the regions defined by the recombinant (effectively, positions 1–618 and 619–1299 in the ex1ex2 alignment) were found to be statistically different in their topologies (SH & AU tests with all p-values < 0.0001). Gene tree reconstruction on the coding dataset, with regions of the T70 *C*. *rhomboideum* recombinant sequence separated, clearly resolved separate *AT3-1* and *AT3-2* clades with 100% confidence. The T70 *C*. *rhomboideum* sequence portion derived from the *AT3-1* parent (effectively bp 1–618) was nested strongly inside *AT3-1*. *Catf2* is completely supported as sister to *AT3-1*. The portion of T70 likely derived from *AT3-2* (effectively bp 619–1299) is resolved as sister to the clade comprising all Solanaceae *AT3-2* sequences with complete support (Fig 4), analogous to the position of *catf2* relative to *AT3-1*.

**Fig 5:**
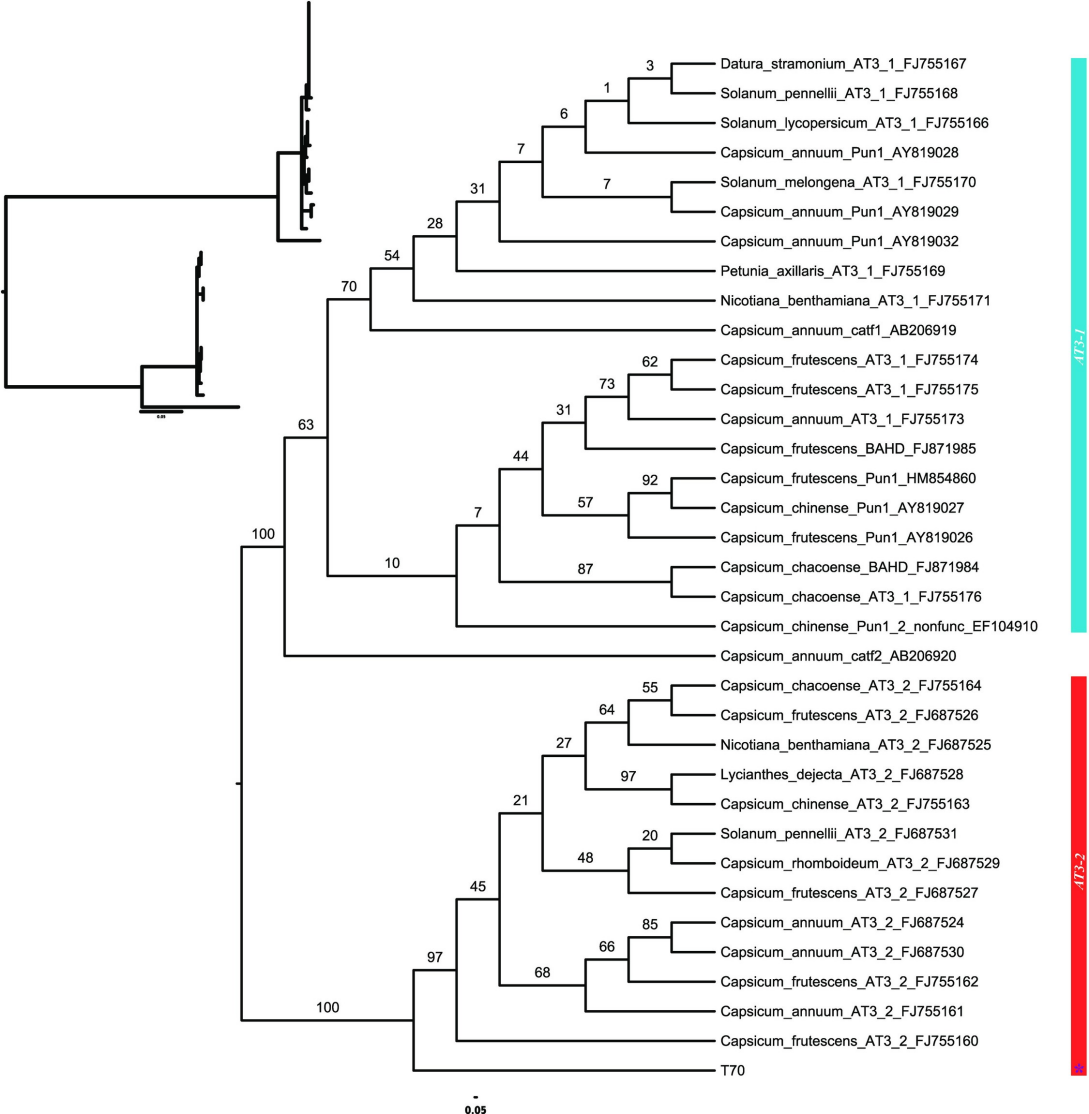
Gene tree estimate from intronic regions using RAxML with 100 bootstrap replicates. Numbers above branches are bootstrap support values for corresponding nodes. Inset shows branch lengths.

Phylogenetic analysis of the intronic regions of *AT3-1* and *AT3-2* across the taxa sampled strongly resolve the paralogs and support the placement of the T70 *AT3-1* intron as sister to the *AT3-2* clade (Fig 5). Gene tree estimation with recombinant regions of T70 *C*. *rhomboideum* separated significantly improved resolution along the backbone and improved nodal support across the tree (Fig 4) relative to the gene tree that was constructed disregarding recombination (Fig 3).

Surprisingly, *AT3-1* and *AT3-2* are not resolved as sister clades, but *AT3-2* is moderately supported (BP = 72) as sister to a clade comprised solely of *Solanum* acylsugar-3-like paralogs. Significant expansions of the acylsugar-3-like gene family within *Nicotiana* and *Solanum* are illustrated here, expansions that likely occurred after the relative establishment of these genera (Fig 4). Maximum likelihood phylogenies of the reduced datasets used for *Ks* estimation for the full coding (ex1ex2br), predominantly exon 1 (ex1br), and predominantly exon 2 (ex2br) data partitions (Figures A-C in S2 File, respectively) were consistent with phylogenies estimated based on the full sampling set (Fig 4).

### *Ks* estimation

**Fig 6:**
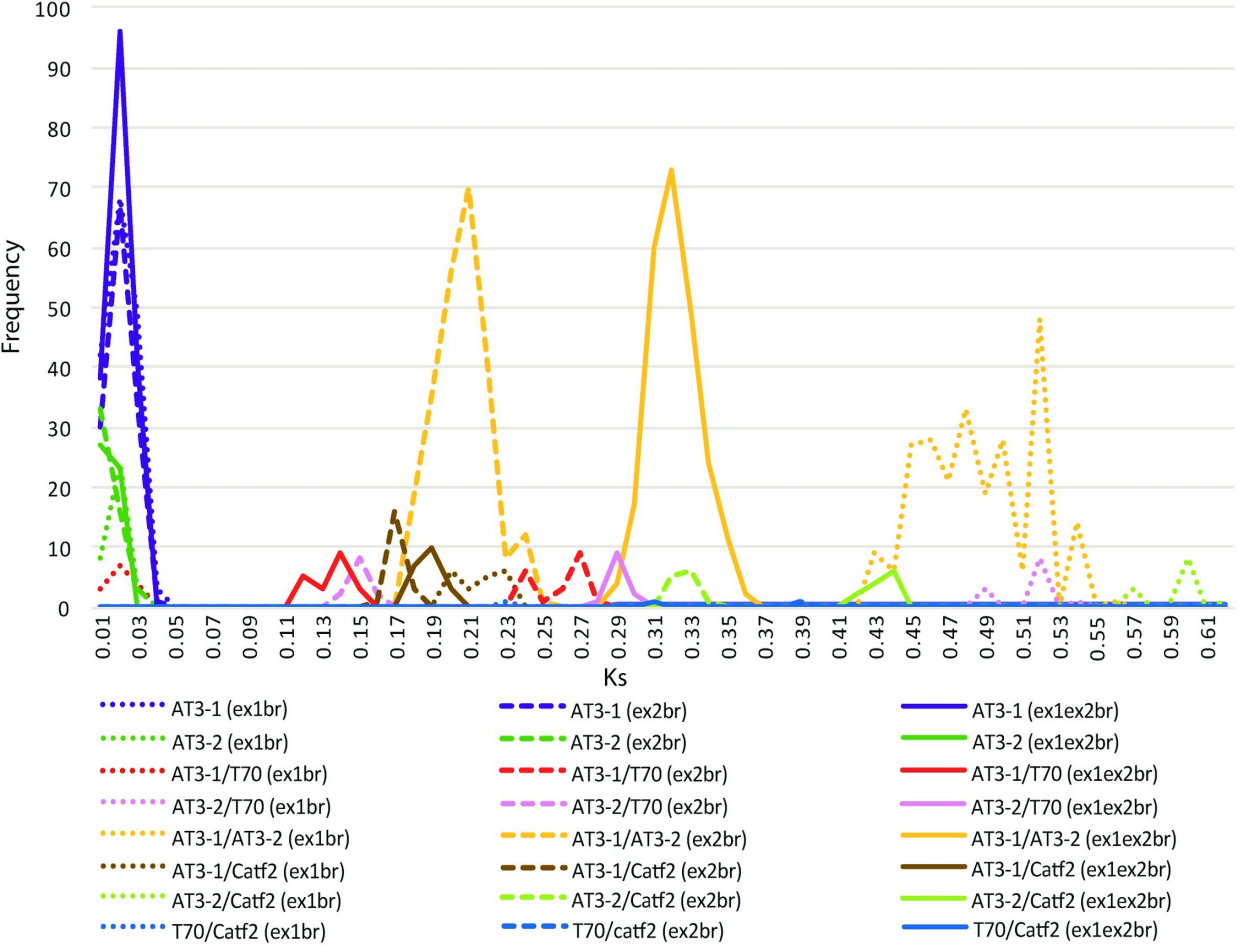
Distribution of Ks estimates from pairwise comparisons sampling across *AT3-1*, *AT3-2*, the recombinant *Capsicum rhomboideum* and *C*. *annuum* acylsugar-3-like genes. Estimates were calculated for the total gene alignment (ex1ex2br) and across each side of the estimated recombination breakpoints (ex1br and ex2br).

Synonymous substitution rates (*Ks*) were estimated for each pairwise comparison within a reduced dataset of 44 sequences (S3 File). Summary statistics for collective pairwise comparisons within and between key clades and by ex1br, ex2br, or ex1ex2br data partitions were computed (S3 File). For the full coding dataset, the average pairwise synonymous substitution rate between *AT3* paralogs was statistically different, even without the T70 recombinant (Table 2). Seen graphically, however, the ranges of *Ks* estimates for *AT3-1* and *AT3-2* overlap (Fig 6). Tukey’s honestly significant difference (HSD) statistics across pairwise comparisons for the breakpoint-partitioned datasets (ex1br and ex2br) by *AT3-1* and *AT3-2* clades finds that means for all comparisons are strongly statistically significant, *except*: *AT3-2* ex1br vs *AT3-1* ex2br, which is barely significant with a p-value of 0.046; and AT3-2 ex1br vs *AT3-2* ex2br which is not statistically different. The rates of evolution within ex1 and ex2 within *AT3-1* are statistically different (Table 3).

**Table 2:** ANOVA test of difference in means across pairwise estimates of Ks within AT3-1 and AT3-2 clades.

SUMMARY						
*Groups*	*Count*	*Sum*	*Average*	*Variance*		
*AT3-1*	175	2.7385	0.0156	3.418E-05		
*AT3-2*	50	0.4764	0.0095	1.829E-05		
ANOVA						
*Source of Variation*	*SS*	*df*	*MS*	*F*	*P-value*	*F crit*
Between Groups	0.001456832	1	0.001456	47.4707	5.6375E-11	3.883
Within Groups	0.006843658	223	3.0689E-05			
Total	0.00830049	224				

SS: sum of squares; df: degrees of freedom; MS: means square; F: F score; F crit: critical F score

**Table 3:** Tukey honestly significant difference statistics for multiple pairwise comparisons across separate breakpoint means and AT3-1 and AT3-2 clades.

Comparison	Tukey HSD Q statistic	Tukey HSD p-value	Tukey HSD inference
AT3-1 ex1br vs AT3-2 ex1br	7.2268	0.0010053	** p<0.01
AT3-1 ex1br vs AT3-1 ex2br	5.5138	0.0010053	** p<0.01
AT3-1 ex1br vs AT3-2 ex2br	10.899	0.0010053	** p<0.01
AT3-2 ex1br vs AT3-1 ex2br	3.6847	0.0468434	* p<0.05
AT3-2 ex1br vs AT3-2 ex2br	1.7841	0.5775785	insignificant
AT3-1 ex2br vs AT3-2 ex2br	6.6613	0.0010053	** p<0.01

*denotes statistical significance at p<0.05;

**denotes statistical significance at p<0.01

In assessing the range of pairwise *Ks* estimates within and between clades (Fig 6), two striking patterns are evident. First, when compared across datasets, the full coding dataset (ex1ex2br) is always intermediate between the range of estimates for the breakpoint partitions. *Ks* ranges afforded by ex1br are always on the higher end, with the exception of *AT3-1*/T70 and T70/catf2 comparisons. Second, the *Ks* rate is higher within ex1br for all clade comparisons, except the *AT3-1*/T70 comparison, than the *Ks* rate apportioned by ex2br. This provides further evidence for the role of *AT3-1* in the recombination event (Fig 6), suggesting either an accelerated rate of neutral evolution in ex1br relative to ex2br in *AT3-1*, or that the ex1 construct within the gene is older than ex2.

## Discussion

### Evolution of the *AT3* gene family

The *AT3* gene family was first linked with the *Pun1* gene in *Capsicum* and shown to be an acyltransferase by Stewart et al. [6]. Since then, studies have discovered gene family expansion within *AT3*. Zhang et al. [11] outlined three main *AT3* lineages incorporating 21 putatitve acyltransferases. Qin et al. [39] outlined three main lineages of tandemly duplicated *AT3* paralogs along with homologous sequences in potato and tomato. Kim et al. [9] detailed seven tandemly duplicated paralogs with sequence similarity to the *AT3* sequences reported in our study, however, this study designated *AT3* as capsaicin synthase. Our sequence analysis and gene tree reconstruction clearly resolve a tandem gene duplication event in the Solanaceae involving the *AT3* locus in the BAHD acyltransferase family, with subsequent pseudogenization of the second locus. Based on the results in this paper, we amend the designation of the functional *AT3* locus previously reported in *Capsicum* to be *AT3-1*, now resolved from the second sequence, *AT3-2*. Without exception, the intron-exon boundary and intronic sequence of *AT3-1* were conserved, an unusual finding for nuclear gene sequences from the Solanaceae [40, 41]. The tight monophyly of the respective *AT3* clades, and their association with some other solanaceous genera including *Datura*, *Petunia*, and *Nicotiana* are consistent with the hypothesis that *AT3-1* and *AT3-2* are ancient paralogs whose duplication predates the speciation of taxa sampled in this study.

Within the *AT3* clades, we determined that relationships are largely unresolved and poorly supported, consistent with a high degree of conservation within clades and across genera. Separate *AT3-1* and *AT3-2* clades were strongly supported by bootstrap analysis. Relationships within clades are less resolved. Lower levels of statistical support were observed within clades, with the exception of the *AT3-1* clade containing the major cultivated *Capsicum* varieties plus *Lycianthes*. In contrast to the other clades, this grouping showed good bootstrap support of 91% (Fig 3). The present analysis supports previous studies that reported close association between the three major cultivated species of *Capsicum*: *C*. *annuum*, *C*. *chinense* and *C*. *frutescens* [40]. Our data also support the close relationship between *Capsicum* and *Lycianthes* [42]. The lack of resolution within clades, however, precludes a conclusive determination as to whether *Capsicum* is nested within a paraphyletic *Lycianthes*, a relationship supported by various phylogenetic studies, e.g. [43–45], or sister to a monophyletic *Lycianthes*, a relationship mostly determined by chloroplast gene regions [45, 46]. However, resolution of one of these two relationships between *Capsicum* and *Lycianthes* are not always the case. In a phylotranscriptomic study of 5,545 genes across six transcriptomes of *Capsicum* and *Lysianthes*, over 26% of the genes resolved gene trees wherein the genera were polyphyletic or paraphyletic with respect to each other [45]. What we learn here is that complex gene family relationships can preclude strong resolution and/or congruence of phylogenies estimated therefrom.

### Recombination as a driver of the evolution of pungency in *Capsicum*

A number of evolutionary processes contribute to gene family evolution, however two particularly powerful forces that underlie evolutionary diversification are gene duplication and genetic recombination. These dynamics both result in rearranged genome segments from non-reciprocal exchange between homologous DNA segments via meiotic crossing over. This study reveals genetic sequences consistent with both these effects in the evolution of *AT3* in the Solanaceae. The fact that *C*. *rhomboideum* has a chromosome number of *2n* = 2x = 26, whereas most other *Capsicum* species have *2n* = 2x = 24 chromosomes [47], is direct evidence of such processes at work within *Capsicum*. Further, Scaldaferro et al. [47] discovered that *C*. *rhomboideum* had the lowest haploid karyotype length and a reduced amount of heterochromatin relative to other congeners. Heterochromatin has been shown to play an important role in maintaining genome integrity through repression of recombination [48]. Perhaps this notable difference of heterochromatin in *C*. *rhomboideum* could either have resulted from or predisposed this taxon to recombination. Further, *AT3-1* is located on chromosome 2, at the end of the long chromosome arm [39]. This region has been shown to have high gene diversity as assessed by synonymous and non-synonymous substitution rates, as well as a region that includes a cluster of putatively positively selected genes [45], findings that further support the notion that recombination in this region would may not be uncommon.

In *C*. *rhomboideum*, our study uncovered a recombination event between paralogs *AT3-1* and *AT3-2*, an event that, given the specific shifts in coding sequence, could account for what appears to be universal non-pungency in this species. Our evidence suggests that the recombination event is likely to have occurred exclusively in *C*. *rhomboideum* after the divergence of this species from other *Capsicum* taxa. The recombinant sequence, herein denoted T70, is composed of an exon 1 mostly derived from *AT3-1* spliced to an intron and exon 2 derived from *AT3-2*. This recombination preserves the open reading frame and occurs before the putative active site of the enzyme, denoted by the sequence HXXXXL, which is changed from the canonical HTTTAL to HTMASL. Furthermore, the sequence of *C*. *rhomboideum AT3-1* appears to be truncated by a two-base pair indel which causes a frameshift mutation leading to a stop codon in the second exon, suggesting that the *C*. *rhomboideum AT3-1* transcript would lack the terminal 70 amino acids, and therefore would likely be non-functional. The indel identified in *C*. *rhomboideum AT3-1*, either alone, or in combination with the recombination event between *AT3-1* and *AT3-2*, may be causal for loss of pungency in this accession, supporting the pivotal role of *AT3-1* in capsaicin biosynthesis [6, 12].

Analyses of synonymous substitutions (*Ks*), coupled with gene trees derived from breakpoint-separated datasets, uncovered a patchwork quilt of sequences, built by various processes at work across the evolutionary history of *Capsicum* and the Solanaceae (Figs 4 and 6). The dual nature of the putative effects of the T70 recombinant suggests different phylogenetic and evolutionary signals within its length. From the perspective of pairwise computational comparisons across *Ks* estimates, exon 1 of T70 (ex1br) should be more similar to *AT3-1* than *AT3-2*. In fact, the means for all pairwise comparisons within the T70/*AT3-1* and *AT3-1* (minus T70) clades are nearly identical (S3 File). This, coupled with the result of the *AT3-1*-derived portion of T70 nesting within *AT3-1*, provides solid evidence for *AT3-1* acting as a major parent in the recombination event. Further, graphical analyses of *Ks* distributions by clade-comparisons supports the notion that the exon 1 derived from *AT3-1* may be the more recently originated portion of the recombinant, and that the exon 1 of the former gene (whose relict comprises predominantly the intron and exon 2 of T70) was overwritten by *AT3-1* (Fig 6).

Recombination detection methods suggested that the *AT3-2 Lycianthes dejecta* sequence was the best pick for minor parent of the T70 recombination event. Indeed, gene tree analyses place the portion of T70 derived from *AT3-2* as sister to remaining *AT3-2* sequences (Fig 4), in support of this conclusion. However, it is interesting to note the relative positions of T70 and *Catf-2* genes within the gene trees: *Catf-2* is sister to remaining *AT3-1* sequences (plus *Catf-1*) whereas exon 2 from T70 is sister to the remaining *AT3-2* sequences, a position analogous to that of *Catf-2* relative to *AT3-1* (Fig 4). Lang et al. [49] described two *Capsicum* acyl-transferase genes, *Catf-1* and *Catf-2*, as key players in the developing placenta of *Capsicum*. This study reported that *Catf-2* (sister to *AT3-1* in our gene trees; see Fig 4) was expressed in both non-pungent and pungent lines, but that *Catf-1* (nested within *AT3-1*, and likely synonymous to it) was only expressed in pungent lines. These analogous placements provide some evidence to suggest that *AT3-2* may not be the minor parent, but that perhaps a defunct *Catf* analog–now extinct or overwritten within this recombination event–is. This idea is supported by the fact that *Lycianthes* was chosen as minor parent–not a *Capsicum* sequence–and *Lycianthes* is supposedly outside and basal to *Capsicum* in phylogenetic analyses (e.g. [42]). If this analogy holds, then we would expect the non-recombinant (original) portions of the T70 gene, represented by the ex2br data partition, to be nearly equal for *AT3-1/Catf-2* (*Ks* = 0.1633) and *AT3-2*/T70 (*Ks* = 0.1425). Indeed this is what we observe (Fig 6 and S3 File). Visually, this can be seen in the relative branch lengths of the sister relationships of the *AT3-1/Catf-2* and *AT3-2/*T70 clades (Fig 4). Taken together, this line of inquiry suggests that multiple, physically overlapping recombination events have impacted the diversification of the *AT3* gene family and may have impacted pungency in *C*. *rhomboideum* and perhaps even the genus.

Unraveling the tangled web of recombination events through time and genic space is no trivial task. Case in point, our results do not preclude the possibility that *AT3-1* is actually the more recently derived paralog—not *AT3-2*. The recombination event in *C*. *rhomboideum* may have paved the way for evolution of pungency through later changes within subclades of *AT3-1*. Careful consideration of the phylogenies computed on breakpoint datasets showed that not all of the sequences within the labeled *AT3-1* clade are pungent: pungency is not known from *Solanum*, *Datura*, *Nicotiana* or *Petunia*, all of which are present in the *AT3-1* clade (Figs 3 and 4). Clearly, not all of the *AT3-1* subclades are pungent. It is interesting to note that when the *C*. *rhomboideum* recombinant is separated by breakpoint, the *AT3-1*-derived portion (ex1br) is nested with non-pungent *Datura* and *Petunia*, albeit in a poorly supported clade, not with its congeners of either known pungency or nonpungency. Our fine-scale recombination analyses of gene evolution involving *AT3* tandem duplicates within the context of the larger BAHD gene family has enabled an improved understanding of the evolution of pungency within *Capsicum*. It has also illustrated the complex array of events that likely impacted the evolution of pungency and has shown us that further study is needed to fully explain its existence.

### The role of recombination and gene duplication in *AT3* evolution: Family wide and beyond

Gene family evolution is often spurred by tandem gene duplication, a process that results in gene family clusters within chromosomes. The BAHD superfamily has been shown to have expanded via tandem gene duplication in *Populus* and *Arabidopsis* [50]. If *AT3-2* resulted from a tandem gene duplication of *AT3-1* alone, we would expect their respective clades to be sister to each other in the gene tree analysis. Instead, we see *AT3-2* sister to a clade comprised solely of *Solanum* sequences, likely representing the expansion of a gene family member following the divergence of *Solanum*. Qin et al. [39] found similar evidence, outlining three main *AT3* lineages, alongside an expansion within *Solanum* (potato and tomato). This suggests that the origin and differentiation of *AT3-2* did not arise from tandem gene duplication alone, but may have also involved a recombination event between older paralogs within the BAHD superfamily.

Kim et al. [9] outlined seven paralogs co-located within a 1-Mb region on an unspecified chromosome classified within two ancient lineages, α and β, that originated via a whole genome duplication in the pepper-tomato ancestor (estimated at a *Ks* of 0.3) [39, 51]. Unfortunately, Kim et al.’s seven *CS* paralogs were not available publicly for incorporation into our study. Because of this, we were not able to definitively determine how our *AT3* lineages aligned with theirs. That said, if we were to suppose our duplication event was captured within Kim et al.’s seven paralogs, we might surmise that our *AT3-1* and *AT3-2* equates to their α and β lineages based on synonymous substitution rates; indeed, we find *Ks* = 0.32 for *AT3-1/AT3-2*, ignoring recombination (ex1ex2br). Assuming a synonymous substitution rate of 6.1 × 10 ^− 9^ substitutions per synonymous site per year (s/s/year) [52]), this would suggest that these genes were duplicated ~26 mya. However, taking recombination into account tells a different story: in looking at all the clade comparisons that cross the *AT3-1/AT3-2* node, *Ks* ranges from 0.48 to 0.59 for exon 1 and from 0.2 to 0.38 for exon 2 (Fig 6 and S3 File). Exon 1 is approximately twice as old as exon 2 based on *Ks* estimates. This data now compounds the complexity of gene duplication with whole genome duplication, and estimating the timing of whole genome duplications can be fraught with difficulties depending on whether it was allo- or autopolyploid [53]. Whole genome duplication is often followed by “genomic shock” in which widespread gene loss and homologous recombination takes place, e.g.[54]. One scenario that might explain our findings would involve an ancient tandem gene duplication event creating the α and β lineages about 39 to 48 mya (*Ks* = 0.48–0.59), the signal for which resides still in exon 1, followed by a whole genome duplication event happening between 31–48 mya, that subsequently went through genomic shock which spurred recombination between the ancient duplicates between 16–31 mya (*Ks* = 0.2–0.38 estimated by exon 2). This hypothesis of an ancient recombination event impacting *AT3* expansion is also supported by the fact that *AT3-1* and *AT3-2* have non-homologous intronic regions.

Recently, the pepper genome v.1.55 was made publically available, detailing a chromosome level assembly for an F1 of a cross between a landrace, Criollos de Morales 334 (CM334) and a non-pungent pepper-breeding line [55]. A BLAST comparison of *AT3-2* from *C*. *annuum* (GenBank number FJ687524) against the pepper genome v.1.55 aligns with 100% id to a region on chromosome 2 corresponding to gene model CA02g19300. In contrast, a BLAST comparison of *AT3-1* from *C*. *annuum* (GenBank number FJ755173) hits with 98.78% identity to a region also on chromosome 2, but corresponding to gene model CA02g19260.

Few studies outside of model organisms have tracked the evolutionary history of a tandem gene duplication event in detail [56,57]. In contrast, most studies of gene duplication events have focused on related organisms for which whole genome sequence is available [58, 59]. Although functional pseudogenes have been identified in the past, evidence linking pseudogenes to their putative functions are still not extremely common [57, 60, 61]. The existence of *AT3-2* was confirmed by screening the sequence against *C*. *annuum* cultivars on a Pepper Chip comprised of over 30,000 unigenes derived from expressed sequenced tags. Extractions of total genomic DNA from 40 *C*. *annuum* cultivars yielded nine unigenes that hybridized with the locus corresponding to *Pun1* (*AT3-1*), two of which had 100% identity to *AT3-2* [62]. In general, *C*. *rhomboideum* is considered non-pungent [45, 47]. The tandem gene duplication event described herein coupled with knowledge of pungency across Solanaceae are consistent with a critical role for *AT3-1* in enabling capsaicinoid biosynthesis, the loss of which in *C*. *rhomboideum* through putative recombination with its tandem, pseudogenized gene duplicate *AT3-2*, likely contributed to non-pungency of this species and may have influenced the course of evolution with regard to pungency in other *Capsicum* species.

### A call to consider complexity: Integrating fine-scale recombination analyses into gene evolution studies

Given the well-documented, quantitative role of *AT3* in pungency, we had assumed sequences from non-pungent Solanaceae taxa would reveal significant differences that might ultimately inform our understanding of the origin of pungency and/or *AT3* function. Instead, the high level of sequence conservation observed, coupled with variable expression patterns and layered with interweaving of phylogenetic signals within and between gene family sequences, has painted a highly complex picture of evolution within the *AT3* gene family, and ultimately the BAHD superfamily. Our work clearly demonstrates the intricate genetic complexity surrounding the production of capsaicinoids and also clearly illustrates the need to incorporate fine-scale analyses of recombination when trying to elucidate gene family evolution. Had we not screened for recombination within our data, a step rarely performed prior to gene tree inferences, our understanding of the evolutionary history of *AT3* evolution within Solanaceae would be far less than afforded by the present work. Further, estimates of *Ks* (and thereby ages of duplication events, be they tandem or whole genome) may be inaccurate when estimated on sequences that harbor unknown recombination events. As such, we sound a clarion call for researchers to incorporate recombination detection tools into their analyses of gene evolution. While doing so may initially reveal patterns that most resemble a crazy quilt of gene duplication and divergence, it will only be through this resolution that the broader patterns of tandem gene duplication, whole genome duplication, recombination, and the structural and functional divergence that underlie the evolution of the staggering array of plant metabolic diversity, including such novel metabolites as capsaicin, may be thoroughly revealed.

## Supporting information

S1 File Reduced alignments used for *Ks* estimation within PAML for 44 sequences parsed by exon 1 and exon 2 and presented in phylip format. (TXT)

S2 File Supplementary figures, including: Figure A–RAxML maximum likelihood phylogeny of the 44-sample Ks ex1ex2 dataset; Figure B–RAxML maximum likelihood phylogeny of the 44-sample Ks ex1br dataset; Figure C–RAxML maximum likelihood phylogeny of the 44-sample Ks ex2br dataset.
(PDF)

S3 File Tables of *Ks* estimates for all pairwise comparisons of the 44 individuals shown in the S1 File, inclusive of multiple sheets for *Ks* estimates from ex1ex2, ex1br, and ex2br. Summary statistics (Sum_stats) are compiled for each data set (Ks_ex1br, Ks_ex2br, Ks_ex1ex2br) with cells in the raw estimates sheets relevant to each comparison color coded to match. The raw data are used to create Fig 6.
(XLSX)
