# Supplementary material for: Tandem gene duplication and recombination at the AT3 locus in the Solanaceae, a gene essential for capsaicinoid biosynthesis in Capsicum
Source: PLoS One. 2019 Jan 23;14(1):e0210510. doi: 10.1371/journal.pone.0210510 (PMC6343889; doi:10.1371/journal.pone.0210510)

Supplementary File 2.

Supplementary Figure A. Raxml maximum likelihood phylogeny using the 44-sample dataset used for *Ks* estimation and using the full complement (ex1ex2) of coding sequence.

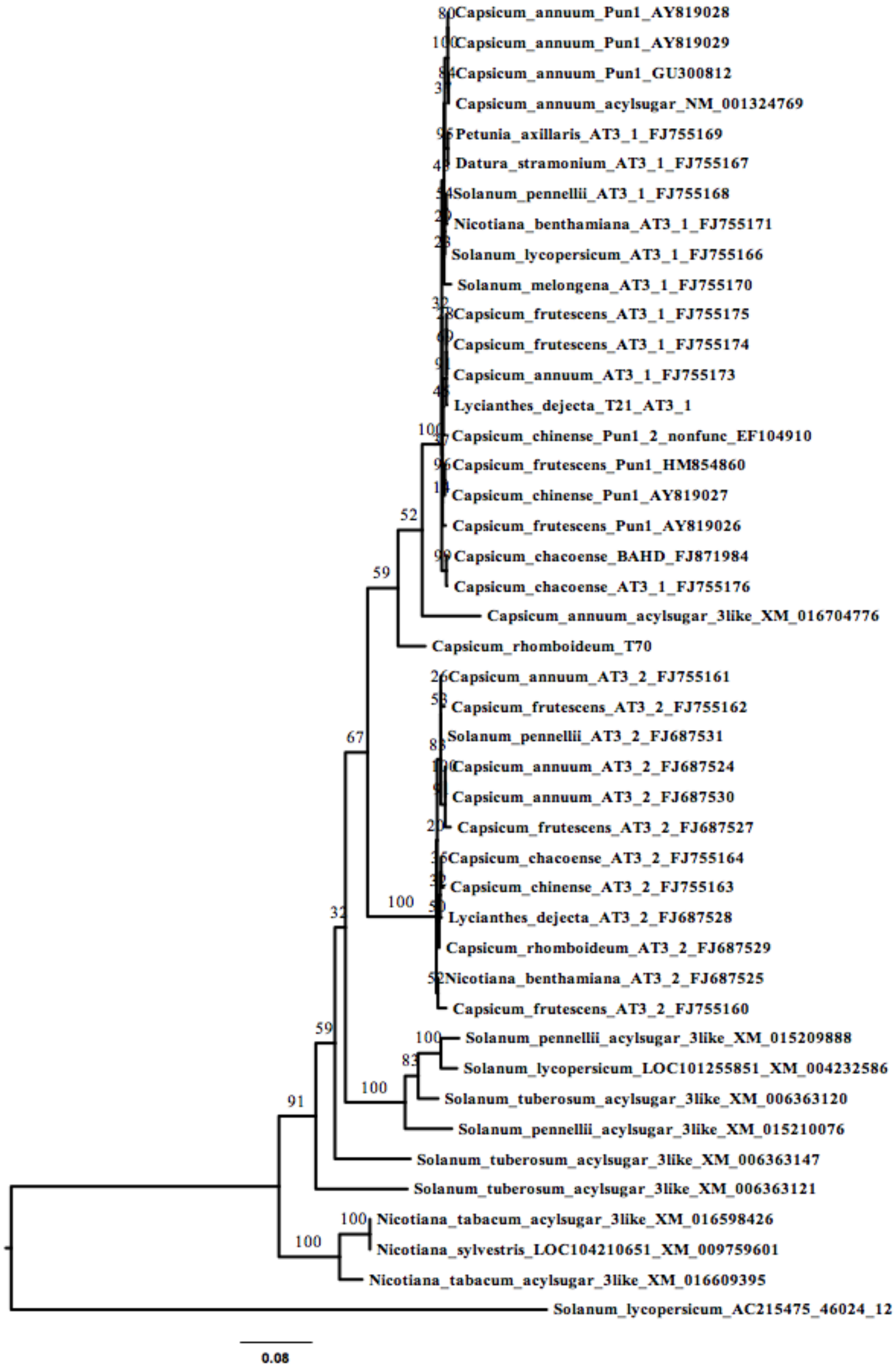

Supplementary Figure B. Raxml maximum likelihood phylogeny using the 44-sample dataset used for *Ks* estimation cut at the breakpoint and incorporating predominantly exon 1 of coding sequence (ex1br).

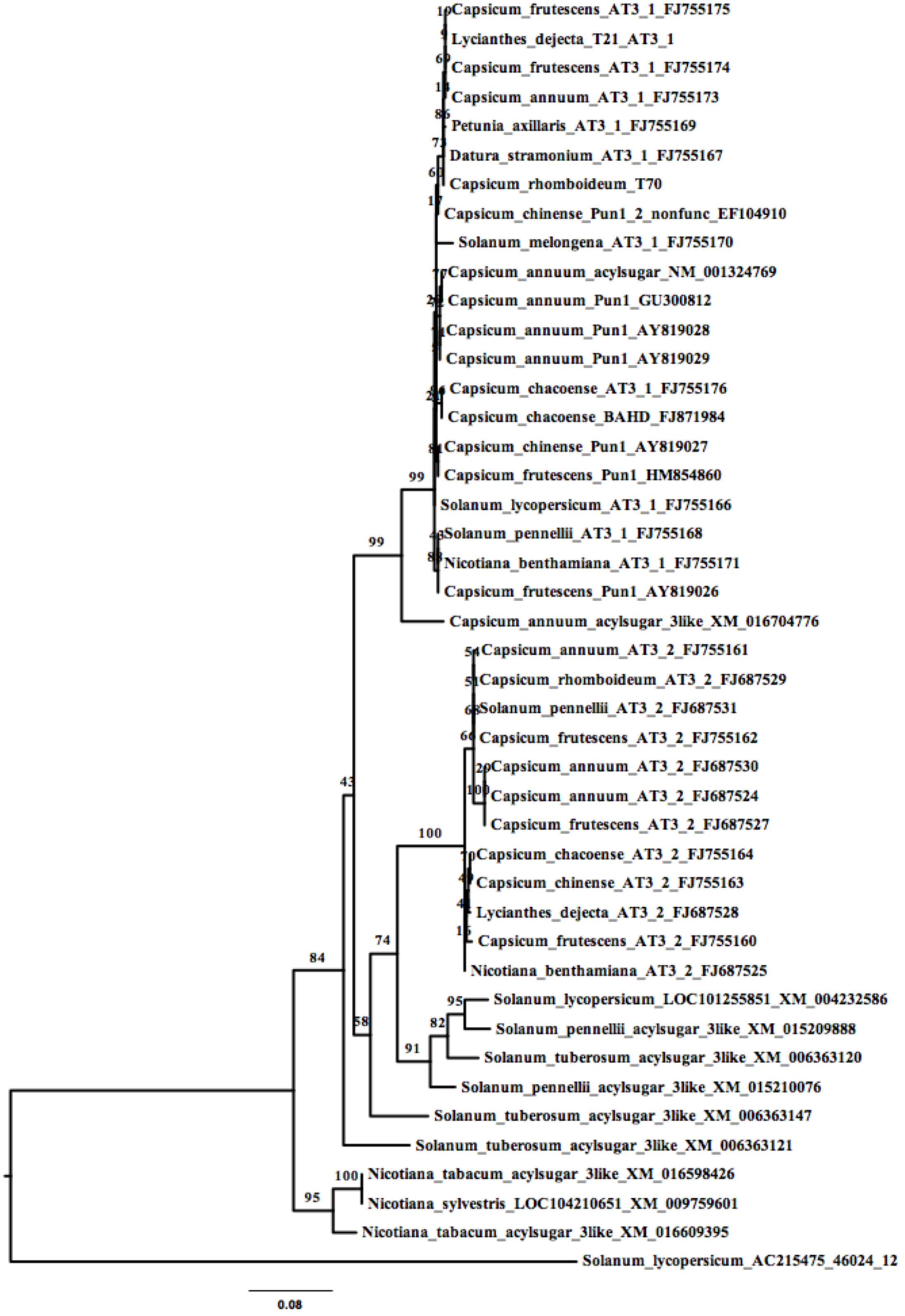

Supplementary Figure C. Raxml maximum likelihood phylogeny using the 44-sample dataset used for *Ks* estimation cut at the breakpoint and incorporating predominantly exon 2 of coding sequence (ex2br).

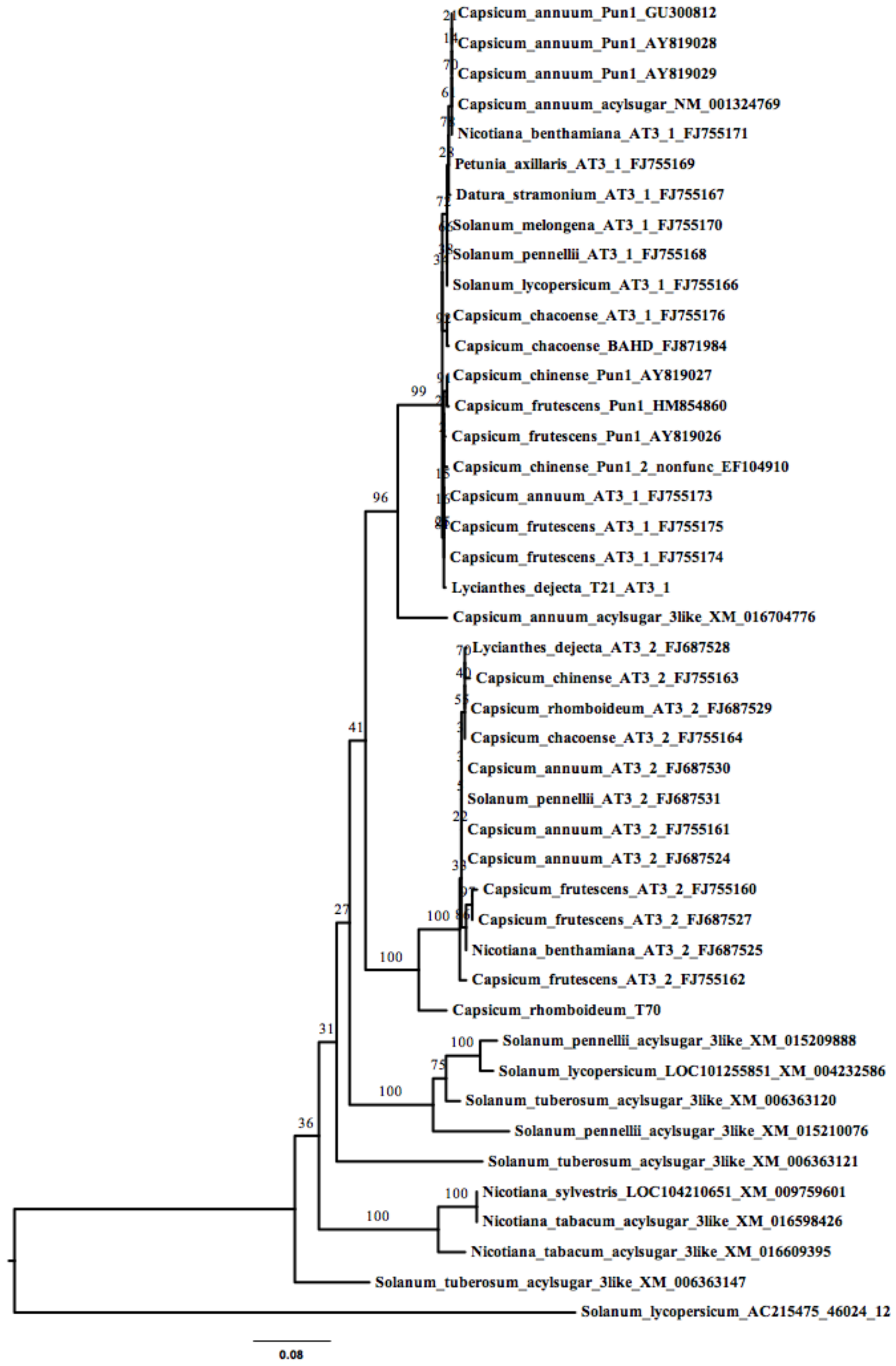

Supplement: S2 File — Supplementary figures, including: Figure A–RAxML maximum likelihood phylogeny of the 44-sample Ks ex1ex2 dataset; Figure B–RAxML maximum likelihood phylogeny of the 44-sample Ks ex1br dataset; Figure C–RAxML maximum likelihood phylogeny of the 44-sample Ks ex2br dataset. (PDF) [file pone.0210510.s002.pdf]
